# Patterns and predictors of off-label prescription of psychiatric drugs

**DOI:** 10.1371/journal.pone.0198363

**Published:** 2018-07-19

**Authors:** Aishwarya Vijay, Jessica E. Becker, Joseph S. Ross

**Affiliations:** 1 Yale University School of Medicine, New Haven, Connecticut, United States of America; 2 MGH/McLean Psychiatry Residency Program, Massachusetts General Hospital, Boston, MA, United States of America; 3 Harvard Medical School, Boston, MA, United States of America; 4 Section of General Internal Medicine and the National Clinician Scholars Program, Yale School of Medicine, New Haven, Connecticut, United States of America; 5 Department of Health Policy and Management, Yale School of Public Health, New Haven, Connecticut, United States of America; 6 The Center for Outcomes Research and Evaluation, Yale–New Haven Hospital, New Haven, Connecticut, United States of America; University of Rome Tor Vergata, ITALY

## Abstract

Off-label prescribing of psychiatric drugs is common, despite lacking strong scientific evidence of efficacy and potentially increasing risk for adverse events. The goal of this study was to characterize prevalence of off-label prescriptions of psychiatric drugs and examine patient and clinician predictors of off-label use. This manuscript presents a retrospective, cross-sectional study using data from the 2012 and 2013 National Ambulatory Medical Care Surveys (NAMCS). The study examined all adult outpatient visits to psychiatric practices for chronic care management with a single listed visit diagnosis in which at least one psychiatric drug was prescribed. The main outcome measure was off-label prescribing of at least one psychiatric drug, defined as prescription for a condition for which it has not been approved for use by the FDA. Among our sample representative of 1.85 billion outpatient visits, 18.5 million (1.3%) visits were to psychiatrists for chronic care management in which at least one psychiatric drug was prescribed. Overall, the rate of off-label use was 12.9% (95% CI: 12.2–15.7). The most common off-label uses were for manic-depressive psychosis treated with citalopram and primary insomnia treated with trazodone. Several patient and clinician characteristics were positively associated with off-label prescribing, including seeing a psychiatrist (OR: 1.06, 95% CI, 1.01–1.12; p = 0.03) instead of another type of clinician, the office visit taking place in the Western region of the country (OR: 1.09, 95% CI, 1.01–1.17; p = 0.02), and the patient having 3 or more chronic conditions (OR: 1.12, 95% CI, 1.02–1.14; p = 0.003). In contrast, having Medicare coverage (OR: 0.93, 95% CI, 0.84–0.97; p = 0.04) and receiving payment assistance from a medical charity (OR: 0.91, 95% CI, 0.88–0.96; p = 0.03) instead of private insurance were negatively associated with off-label prescribing. These results suggest that certain classes of psychiatric medications are being commonly prescribed to treat conditions for which they have not been determined by the FDA to be clinically efficacious and/or safe.

## Introduction

Off-label prescribing is the prescription of an FDA-approved medication for a condition or in a manner different from that approved by the FDA. This practice is legal and common–a 2003 report showed that for the 3 leading drugs in each of the 15 leading drug classes, off-label use accounted for approximately 21% of prescriptions [1]. Off-label prescribing does have potential benefits in certain situations. It encourages innovation in clinical practice and allows approved therapies to be used for rare conditions that have not been as well studied. Nonetheless, the lack of FDA approval for the specific uses means that these drugs have not been subject to the same scientific and regulatory scrutiny as the labeled uses, even if some studies for that indication have been performed. While absence of regulatory approval in and of itself does not mean a drug is harmful in that circumstance, evidence of a drug’s safety and efficacy in one clinical situation may not apply to others [2–5]. In fact, multiple studies comparing adverse drug events among approved vs. off-label uses have found that adverse drug reactions occur at a higher rate among those prescribed for off-label uses [6–9].

Prior research suggests that the highest rates of off-label prescribing are for psychiatric drugs [4, 5, 10], although these studies tend to focus on only one medication, or condition of use [10], or on a specific patient population, such as nursing home residents, Medicaid recipients, or veterans [11–13]. In psychiatry, many medications are prescribed off-label for common conditions that have multiple FDA-approved options already. This may be due to the presumed equivalence of various medications within a class, e.g. the substitution of one selective serotonin reuptake inhibitor (SSRI) for another for treatment of depression, without necessarily evidence of efficacy. A recent document from the ADAA (Anxiety and Depression Association of America), for instance, shows that several of the medications commonly prescribed for various anxiety disorders do not actually have an FDA-approved indication but are within the same class as one that has an FDA approval for the indication [14]. In some cases, this may be justifiable–for example, escitalopram is approved for treatment of generalized anxiety disorder, but its enantiomer, citalopram, is not [15]. Nevertheless, in a study surveying off-label antidepressant prescription in primary care, 84.2% of off-label prescriptions had no strong evidence of efficacy for the indication [16]. Of this, 45% of prescriptions were for a class of drugs where no drug in the class had strong evidence of efficacy [16]. Other studies suggest that 20% of total drug sales for treatment of insomnia drugs are for anti-depressants, despite weak evidence of the effectiveness of antidepressants as primary treatment for insomnia patients [17–19].

Physicians’ reasons for prescribing off-label treatment are often difficult to discern, even after reviewing electronic medical records [20]. Physicians may erroneously believe that the medications are safe and efficacious for an off-label use, or they may not be aware of the FDA-approved indication for use [21]. In order to understand and address the high levels of off-label prescription in the United States, it is important to examine predictors of off-label use using a nationally representative sample of prescription practices. Accordingly, our main objective was to characterize prevalence of both on-label and off-label use of four commonly prescribed classes of psychiatric drugs–antipsychotics, antidepressants, stimulants and anxiolytics–using a cross-sectional sample from a nationally representative database of office visits to nonfederal clinics in the United States. In addition, we examined patient and prescriber predictors of off-label use.

## Methods

### Data source

Data were extracted from the 2012 and 2013 National Ambulatory Medical Care Survey (NAMCS), the most recent period for which these data were available. The survey is an ambulatory component of the National Health Care Survey conducted by the US National Center for Health Statistics (NCHS, a division of the Centers for Disease Control and Prevention). NAMCS samples non-federally employed, office-based healthcare providerswho are primarily engaged in direct patient care. This does not include providers in the field of anesthesiology, radiology or pathology. The data provide an analytic base that serves as an important tracking tool on ambulatory care utilization regarding national trends, medication use, and practice patterns in the US [22].

The NAMCS collects information about patients' office visits. The basic sampling unit is the physician-patient encounter or outpatient visit. The data contain information about patients' demographic characteristics (i.e., age, race, gender, insurance type, and region), up to three diagnoses, and up to ten records of prescription and non-prescription medications for each visit. The study was approved by the Institutional Review Board of Yale University.

All NAMCS data were fully anonymized before they were accessed for use in this study. IRB approval, licensing, patient and provider consent was all performed by the National Center for Health Statistics previous to release of the survey data (IRB# 2016–03). The survey sample was composed of randomly selected physicians based on information obtained from the American Medical Association (AMA) and the American Osteopathic Association (AOA). Participants were asked to provide data on approximately 30 patient visits during a randomly assigned 1-week reporting period.

### Study sample

**Fig 1:**
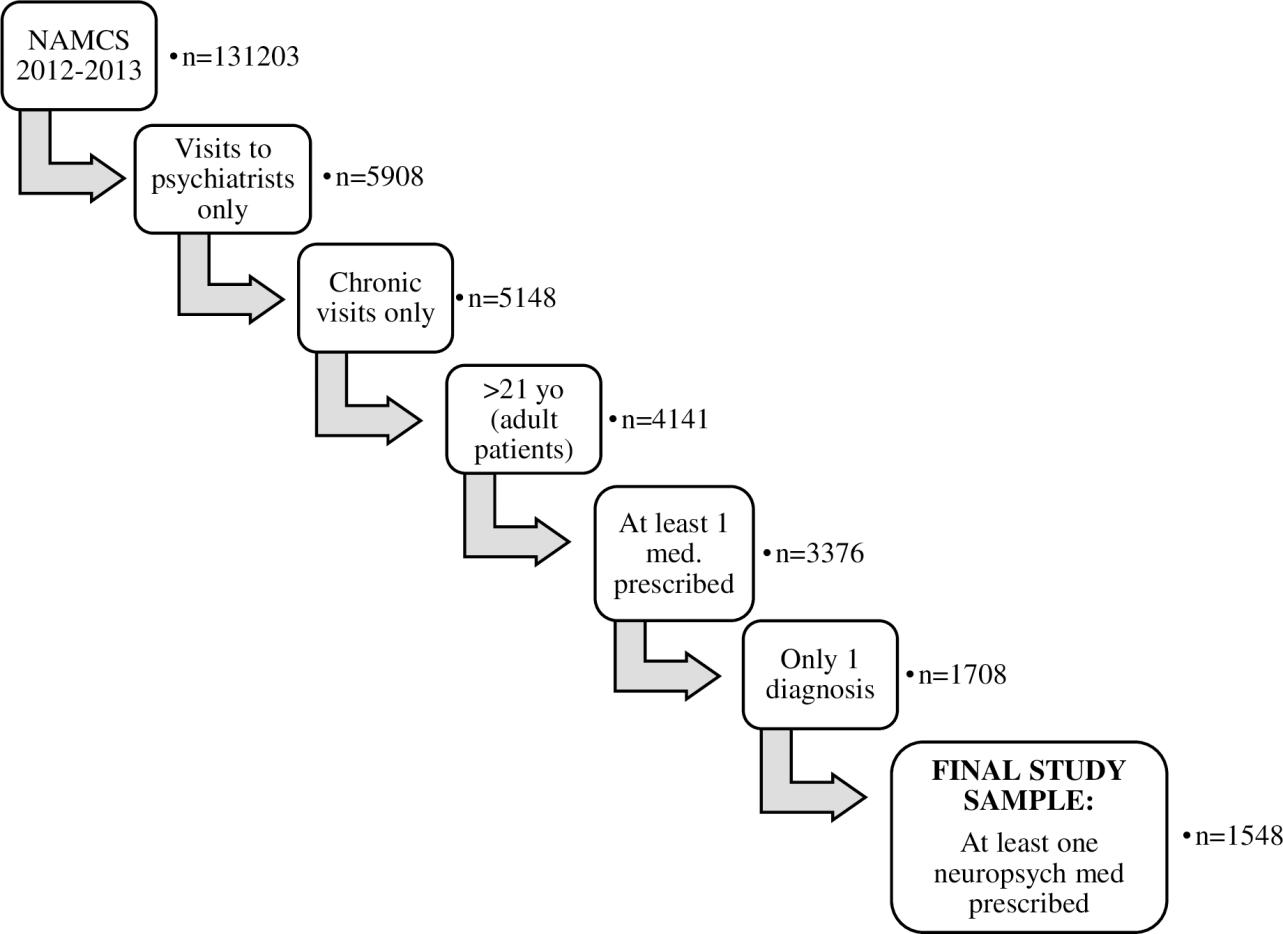
Study sample flowchart.

The study sample was designed to estimate the prevalence of off-label use as conservatively as possible (Fig 1). The sample was first limited to all office visits to a psychiatrist, physician assistant (PA) or psychiatric nurse practitioner (NP)for chronic care management, reflecting the categorization done by NAMCS to assign visits as representing acute care, pre-operative care, preventive care, or chronic care management. The sample was then limited to adult patients 21 years of age or older, to align with FDA pediatric age guidance, [23] who were prescribed at least 1 medication. We subsequently limited the sample to patients for whom only 1 diagnosis was listed as associated with the office visit, to ensure that any determination of off-label use was not a result of there not being space on the survey form to list an on-label indication for use. Finally, we limited the sample to patients who were being treated with at least 1 of 4 classes of drugs: antidepressants, antipsychotics, anxiolytics, or stimulants, among the most commonly used psychiatric drugs in the U.S. We chose not to study mood stabilizers as they had been approved for multiple uses, some out of the realm of psychiatric disorders, at the time of data collection [24].

### Main outcome variable

The main outcome (dependent) variable was whether a patient visit resulted in a prescription for any of the 4 classes of psychiatric drugs for an off-label indication. Up to ten prescriptions, new and continuing, are recorded for each visit in the NAMCS. Drug entries in NAMCS were classified using Multum’s Lexicon Plus system, [25] where each drug was assigned a unique “generic drug code” which was used to classify drug entries in the NAMCS. Within this sample of psychiatric prescriptions, visits in which patients were assigned a diagnosis of a mental disorder (ICD-9-CM codes 290–319, 327, 347) that at least one of the prescribed drugs was approved to treat were classified as being on-label, with the exception of patients with alcohol or drug dependence disorders (ICD-9-CM codes 291–292, 303–305), dementia (290), autism and pervasive developmental disorders (299), speech and language disorders (315–316), and intellectual disabilities (317–319). Off-label uses were determined based on the FDA approved indications for each medication during the time of the study analyses (2012–2013).

### Main independent variables

We examined whether nine patient and clinician characteristics were associated with off-label use, selected based on limited previous work [11, 26] and availability from the data source. Patient characteristics were defined as demographic characteristics (age, sex, and race), payment source (Medicare, Medicaid, private insurance, self-pay, or charity), and medical characteristics (chronic co-morbidities and tobacco use). Patient age was defined categorically as 21–40 years of age, 41–65 years of age and >65 years of age, whereas patients’ chronic co-morbidities were defined as the total number of chronic conditions noted within NAMCS. Prescriber characteristics included state and region the patient was seen in, whether the clinic was in a metropolitan statistical area, and type of clinician seen (MD, PA, NP or otherwise).

### Statistical analysis

In order to obtain nationally representative estimates, sample weights and standard error corrections were incorporated into all analyses. First, a series of descriptive analyses were performed to characterize the demographics of the sample as well as to estimate the survey-weighted frequency of each drug prescription. Next, a weighted multivariate logistic regression was performed to examine predictors of off-label psychiatric drug use. All covariates were evaluated for multi-collinearity; any variable exceeding a variance inflation factor of 7 was removed from the model [27]. A two-tailed statistic with a *P*-value less than 0.05 was considered statistically significant. A Hosmer-Lemeshow goodness-of-fit test was performed to evaluate the fit of the logistic regression model [28]. Survey weights are provided to enable extrapolation of the data to a nationally representative estimate, and were taken into account during data analysis [29]. All data management and analyses were conducted in R Studio version 3.2.3 [30].

## Results

### Participant characteristics

At least one medication was prescribed in 81.5% of all of the adult psychiatric outpatient visits for a chronic issue surveyed in 2012–2013. Of patients who received at least one prescription, 52.8% had only one listed diagnosis. Of these, 91.1% received at least one prescription for one of the four psychiatric drug classes (n = 18,511,829)–this was the sample used for all analyses. Overall, the sample represents 1.3% (18.5 million) of all estimated outpatient visits (both psychiatric and non-psychiatric) in 2012–2013 (1.85 billion).

The majority of visits were made by women (60.0%) and by adults who were predominantly white (91.1%) (Table 1); mean age was 49.0 years (SD = 15.7) and 17.0% were over the age of 65. The most common visit coverage was private insurance (41.3%), followed-by Medicare (21.4%), and Medicaid/CHIP (11.6%). Nearly all visits took place in urban areas (97.7%), as defined by the Metropolitan Statistical Area.

**Table 1:** Socio-demographic characteristics of sample (N = 1548/n = 18,511,829 (weighted)).

Characteristics	N (%) or Mean (SD)*	Off-Label*	On-Label*	p-value
Age (years)–Mean (SD)	49.0 (15.7)	49.6 (16.4)	48.9 (15.5)	0.60
21–40	497 (32.1%)	58 (11.7%)	439 (88.3%)	
41–65	929 (60.0%)	127 (13.7%)	802 (86.3%)	
>65	263 (17.0%)	33 (12.5%)	230 (87.5%)	
Gender				
Female	929 (60.0%)	108 (11.6%)	821 (88.4%)	0.28
Male	619 (40.0%)	91 (14.7%)	528 (85.3%)	
Race				
White	1410 (91.1%)	182 (12.9%)	1228 (87.1%)	0.03^§^
Black	88 (5.7%)	14 (15.8%)	74 (84.2%)	
Other	50 (3.2%)	3 (6.8%)	47 (93.2%)	
Insurance Type				
Private	639 (41.3%)	70 (10.9%)	569 (89.1%)	<0.001^§^
Medicare	316 (20.4%)	44 (13.8%)	272 (86.2%)	
Medicaid/CHIP	180 (11.6%)	17 (9.5%)	163 (90.5%)	
Charity/No Charge	168 (10.8%)	11 (6.8%)	157 (93.2%)	
Self-Pay	142 (9.2%)	22 (15.6%)	120 (84.4%)	
Other	77 (5.0%)	5 (6.0%)	72 (94.0%)	
Unknown	26 (1.7%)	4 (17.0%)	22 (82.3%)	
Geography				
Region				
Northeast	440 (28.4%)	44 (10.0%)	396 (90.0%)	0.25
South	435 (28.1%)	62 (14.2%)	373 (85.8%)	
Midwest	359 (23.2%)	40 (11.1%)	319 (88.9%)	
West	314 (20.3%)	53 (17.0%)	261 (83.0%)	
Metropolitan Statistical Area Status (MSA)^†^	1512 (97.7%)	194 (12.8%)	1318 (87.2%)	0.71
Type of Clinician Seen				
Psychiatrist	1379 (89.1%)	245 (17.8%)	1134 (82.2%)	<0.001^§^
Other	169 (10.9%)	18 (10.6%)	151 (89.4%)	
Current Tobacco User	149 (9.6%)	11 (7.6%)	138 (92.4%)	0.04^§^
# of Chronic Conditions^‡^				
0	512 (33.1%)	68 (13.3%)	444 (86.7%)	<0.001^§^
1	834 (53.9%)	105 (12.6%)	729 (87.4%)	
2	152 (9.8%)	20 (13.1%)	132 (86.7%)	
3+	50 (3.2%)	9 (18.1%)	41 (81.9%)	

* n is an un-weighted estimate; Off-label sample size is N = 216/n = 2381110 and on-label sample size is N = 1332/n = 16130719. % correspond to the row category.

^†^ OMB defines a Metropolitan Statistical Area as one or more adjacent counties) or county equivalents that have at least one urban core area with a population of at least 50,000, plus adjacent territory that has a high degree of social and economic integration with the core as measured by commuting ties

^‡^ NAMCS has 14 different chronic conditions that can be checked off by the physician: Arthritis, Asthma, Cancer, Cerebrovascular Disease, COPD, Chronic Renal Failure, CHF, Depression, Diabetes, Hyperlipidemia, Hypertension, Ischemic Heart Disease, Obesity, and Osteoporosis

^§^ p<0.05 indicates a significant difference between the two groups

### Psychiatric medications

**Fig 2:**
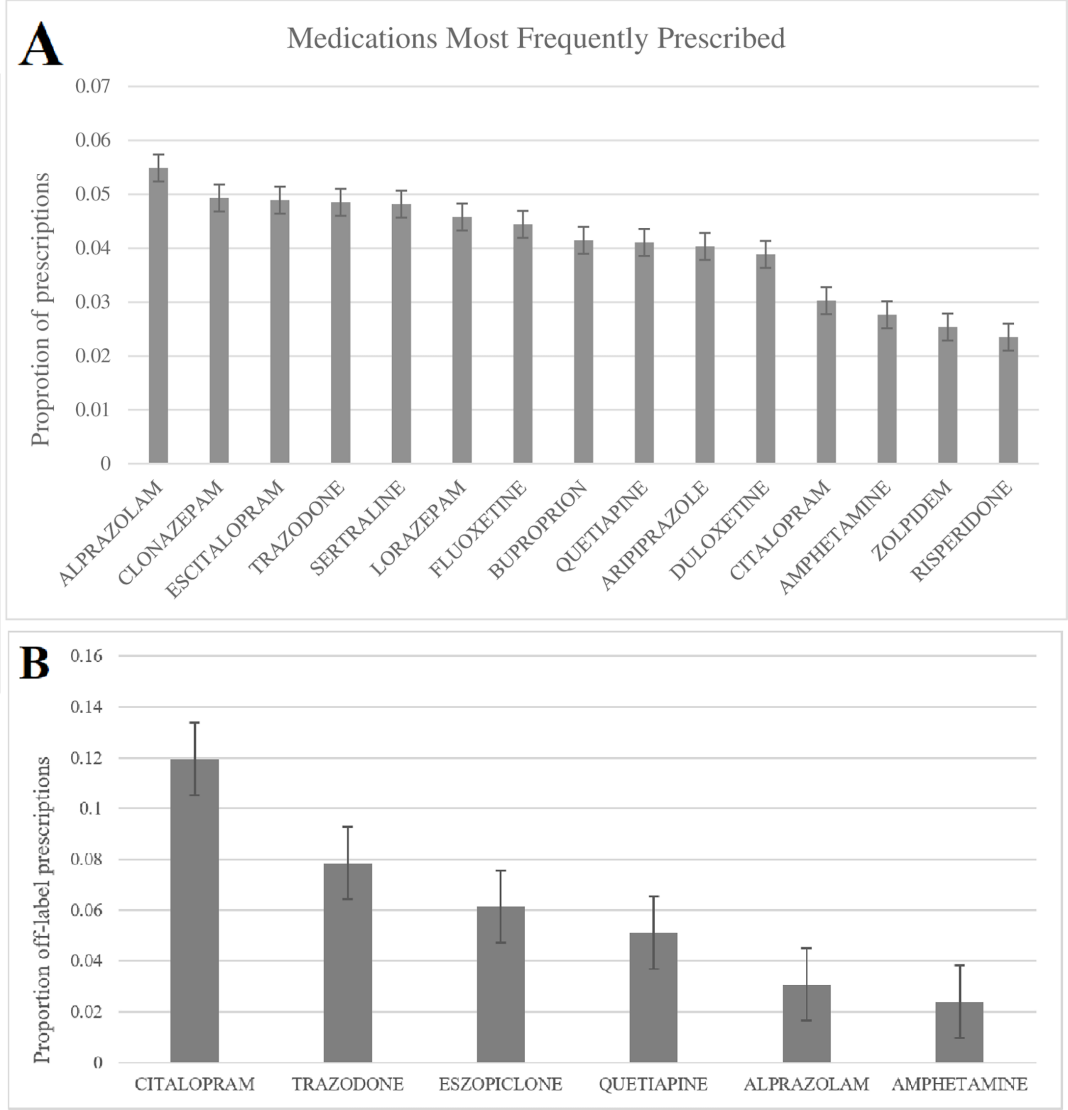
Medication characteristics. (A) Most Frequently Prescribed Medications (N = 1548/n = 18,511,829) (B) Most Frequently Prescribed Off-Label Medications (N = 216/n = 2381110).

The median number of psychiatric medications prescribed per patient visit was 2 (IQR = 1–3) (Table 2). The top three most frequently prescribed drugs were alprazolam (5.5% of total psychiatric prescriptions), followed by clonazepam (4.9%), and escitalopram (4.8%) (Fig 2A).

**Table 2:** Medication characteristics (N = 1548/n = 18,511,829 (weighted)).

	Weighted % (95% CI) per group		
Characteristics	Total Sample	Off-Label	On-Label
Median Prescriptions (IQR; Range)*	**2** (2; 1–3)	**2 **(2; 1–3)	**2 **(2; 1–3)
One Prescription Listed	28.1%(25.4–31.0)	5.7% (2.9–6.0)	94.3% (93.6–97.1)
Two Prescriptions Listed	32.8% (29.4–34.3)	4.9% (4.1–7.0)	95.1% (93.0–96.5)
Three Prescriptions Listed	19.4% (18.9–23.0)	4.8% (2.3–5.2)	95.2% (94.8–97.7)
>Four Prescriptions Listed	19.7% (12.4–24.5)	2.8% (0.1–8.0)	97.2% (91.3–99.8)

*The difference in median prescriptions between the off-label and on-label groups was not significant (p = 0.68)

### Off-label use

Overall, 12.9% (n = 2,381,110; 95% CI: 12.2–15.7) of patient visits resulted in an off-label prescription of one of four select medication classes, which did not differ based on the number of psychiatric medications prescribed (p = 0.68; Table 2). Stimulants had the highest rate of being prescribed for an off-label indication (17.6%; 95% CI: 14.3–22.6), followed by anti-psychotics (17.4%; 95% CI: 14.2–21.6), anti-depressants (11.8%; 95% CI: 7.5–15.3), and then anxiolytics (6.7%; 95% CI: 4.6–12.3). However, because anti-depressants were the mostly commonly prescribed, anti-depressants comprised the majority of the prescriptions for off-label use (52.2%; 95% CI: 49.8–55.3).

The most common off-label indications for which drugs were prescribed are presented in Table 3. Citalopram and trazodone had the top two rates of off-label use (Fig 2B). For citalopram, the majority of off-label use was for manic-depressive psychosis (75.9%; 95% CI: 73.2–77.5). For trazodone, the majority of off-label use was for insomnia (54.8%; 95% CI: 47.3–59.8) and anxiety disorders (45.0%; 95% CI: 36.6–52.4). Table 3 also describes the off-label uses for other commonly prescribed off-label drugs.

**Table 3:** Most common diagnoses for commonly prescribed off-label medications.

Medication	On-Label		Off-Label	
	Diagnosis	% (95% CI)*	Diagnosis	% (95% CI)*
Citalopram	MDD-single episode	42.5% (38.3–47.3)	Manic-Depressive psychosis, unspecified	75.9% (73.2–77.5)
	Depression, unclassified	35.6% (30.3–42.1)	Generalized Anxiety Disorder	20.3% (14.7–25.6)
	MDD–recurrent	21.9% (18.2–28.1)	Bipolar affective disorder, mixed	3.4% (1.8–6.2)
			Other	0.4% (0.1–0.6)
Trazodone	MDD-recurrent	35.8% (31.3–39.6)	Insomnia, unspecified	54.8% (47.3–59.8)
	MDD–single episode	33.4% (28.7–37.5)	Generalized Anxiety Disorder	34.3% (28.5–38.1)
	Depression, unclassified	30.8% (23.1–35.2)	Anxiety State, unspecified	10.7% (8.1–14.3)
			Other	0.2% (0.1–0.5)
Eszopiclone	Organic insomnia	96.8% (95.4–97.8)	Bipolar I Disorder	73.2% (68.4–76.6)
	Insomnia, unspecified	3.2% (1.4–7.4)	Manic-Depressive psychoses	26.8% (22.4–29.5)
Quetiapine	Bipolar Mania	43.8% (37.3–49.6)	MDD	51.2% (47.8–54.2)
	Bipolar Depression	31.2% (24.3–38.6)	Generalized Anxiety disorder	40.3% (37.8–42.9)
	Simple type Schizophrenia, chronic	25.0% (21.3–29.4)	Obsessive-Compulsive Disorder	8.5% (6.1–10.3)
Alprazolam	Generalized anxiety disorder	48.6% (43.1–54.3)	Major Depressive Disorder	33.6% (28.6–35.7)
	Panic Disorder	37.1% (34.2–43.7)	Insomnia	28.1% (24.9–33.7)
	Anxiety State, unspecified	14.3% (10.3–18.7)	Neurotic Depression	22.4% (19.4–26.3)
			Prolonged Post-Traumatic Stress Disorder	15.9% (14.7–18.2)
Amphetamine	Attention-Deficit Hyperactivity Disorder	100% (100.0–100.0)	Major Depressive Disorder, single episode	79.2% (76.4–83.2)
			Neurotic Depression	20.8% (17.5–22.4)

* % is weighted

### Predictors of off-label prescribing

Several patient and clinician characteristics were associated with off-label prescribing in multivariate regression analyses. A Hosmer-Lemeshow test showed no evidence of poor fit (χ^2^ = 16.3; p = 0.38). Seeing a psychiatrist (rather than a PA, NP or other type of clinician) (OR: 1.06, 95% CI: 1.01–1.12; p = 0.03), the office visit taking place in the Western region of the country (OR: 1.09; 95% CI: 1.01–1.17; p = 0.02), and having 3 or more non-psychiatric chronic conditions (OR: 1.12; 95% CI: 1.02–1.14; p = 0.003) were positively associated with receiving an off-label prescription (Table 4). In contrast, having coverage with Medicare (OR: 0.93; 95% CI: 0.84–0.97; p = 0.04) and receiving payment assistance from a medical charity (OR: 0.91; 95% CI: 0.88–0.96; p = 0.03) were negatively associated with receipt of an off-label prescription.

**Table 4:** Bivariate and multivariate logistic regressions with off-label use as dependent variable.

		Bivariate Regression			Multivariate Regression		
Explanatory Variable		OR	95% CI	p	OR	95% CI	p
**Patient Characteristics**							
Sex		1.02	-0.98–1.06	0.28	0.2	0.1–0.4	0.27
Race							
	White						
	Black	1.01	0.94–1.08	0.86		1.02	0.94–1.12	**0.24 **
	Other	0.94	0.87–1.02	0.12	0.93	0.86–1.01	0.68
Age							
	21–40 yoa*						
	41–65 yoa	1.01	0.97–1.05	0.51	1.01	0.96–1.07	0.41
	>65 yoa	1.00	0.95–1.06	0.86	1.00	0.91–1.10	0.79
Insurance Type							
	Private*						
	Medicare	0.89	0.76–0.95	0.03†	0.93	0.84–0.97	0.04†
	Medicaid/CHIP	0.96	0.91–1.02	0.24	0.98	0.91–1.05	0.89
	Self-pay	1.02	0.98–1.07	0.35	1.04	0.98–1.10	0.69
	Charity	0.87	0.71–0.93	0.02†	0.91	0.88–0.96	0.03†
	Other	0.96	0.85–0.99	0.04†	0.96	0.88–1.04	0.33
	Unknown	0.97	0.88–1.08	0.60	1.05	0.95–1.15	0.53
Chronic Conditions							
	1*						
	2	1.04	0.95–1.06	0.21	1.01	0.93–1.10	0.13
	3 or more	1.13	1.05–1.19	0.001†	1.12	1.02–1.14	0.003†
Smoker		1.04	0.98–1.11	0.18	1.05	0.98–1.12	0.18
**Clinician Characteristics**							
Clinic in MSA		0.98	0.91–1.07	0.71	1.03	0.96–1.11	0.96
Type of Clinic							
	Psychiatrist	1.07	1.02–1.15	0.03†	1.06	1.01–1.12	0.03†
	Other*						
Region of Clinic							
	Northeast*						
	Midwest	1.01	0.95–1.07	0.79	1.02	0.96–1.08	0.85
	South	1.04	0.98–1.10	0.18	1.06	0.98–1.13	0.26
	West	1.09	1.02–1.13	0.01†	1.09	1.01–1.17	0.02†

* Reference Variable (values for Bivariate is 0 and for Multivariate is 1)

^**†**^ p<0.05; significant variables are also in bold

## Discussion

In our study of a nationally representative outpatient sample of psychiatric visits in 2012 and 2013 during which one of 4 psychiatric medication classes was prescribed, we found that just more than 1 in 8 prescriptions were for an indication not approved by the FDA. There were both patient and clinician characteristics that were positively associated with off-label use. These results suggest that certain classes of psychiatric medications are being commonly prescribed to treat conditions for which they have not been determined by the FDA to be clinically efficacious and/or safe.

We deliberately estimated off-label use conservatively by limiting our sample to chronic care management visits among adult psychiatric patients with a single diagnosis associated with the visit. Nevertheless, almost 13% of visits involved a prescription for off-label use. While this rate is lower than estimates made in other comprehensive off-label studies [4], there is still reason for concern. The drugs with the highest rate of off-label prescription–citalopram and trazodone–were most commonly prescribed as mono-therapeutic off-label treatments for manic-depressive psychosis (citalopram) and insomnia and anxiety (trazodone). The overall body of evidence on the use of antidepressant monotherapy to treat patients with bipolar depression is contentious and not supported by evidence-based guidelines [31]. Furthermore, the efficacy of trazodone in the treatment of primary insomnia or generalized anxiety disorders alone is weak, and the use of certain anti-depressants over FDA-approved benzodiazepines in anxiety disorders has been shown to be associated with a higher risk of adverse drug events [18, 32]. Our findings echo those of a study focusing on insomnia prescriptions that showed 20% of total drug sales in 2006 were for non-evidence-based treatment of insomnia drugs with anti-depressants [17, 18]. Anti-psychotics had the second highest rate of off-label prescription (28.6%)–the most common off-label use was for major depressive disorder. Of note, antipsychotics are being increasingly prescribed as adjunctive therapy for depression and the antipsychotic aripiprazole is FDA-approved as adjunctive depression treatment [33, 34]. Nonetheless, there is little evidence supporting effectiveness of off-label antipsychotic medication as monotherapy for depression and PTSD [35], and antipsychotic medications carry significant risk of adverse effects. Thus, while the rate of off-label use was not inordinately high, the results show that neuropsychiatric medications are still being prescribed to treat conditions for which they have not been found by the FDA to be clinically efficacious and/or safe.

The results of multiple logistic regression indicated that patient and prescriber characteristics were associated with off-label use. With respect to patient characteristics, the study found that having 3+ chronic conditions was positively associated with off-label use. This has been shown in other studies and might be the result of patients with chronic conditions having somewhat refractory disease where multiple therapeutics have been tried, leading to a higher probability that one or more of the treatment choices is an off-label prescription. In contrast, Medicare coverage and receiving payment assistance from a medical charity were found to be negatively associated with off-label prescription use, a finding in part described previously [11]. This could be due to a higher level of formulary management in Medicare plans when compared to other forms of insurance. It is interesting that while older patients with Medicare are more likely to have multiple comorbidities, these two characteristics (Medicare versus comorbidities) are associated with off-label use in opposite directions. It suggests that a very specific patient population–sicker patients with multiple comorbidities that do not receive public insurance–are among those most like to receive prescriptions for off-label uses.

Being seen by a psychiatrist, rather than a PA, NP or other form of medical provider seeing patients in a psychiatric clinic, was positively predictive of off-label use. This finding has not been reported previously, and is potentially an effect of pharmaceutical marketing, wherein psychiatrists with more exposure to marketing may have more comfort using prescription drugs off-label. In the past years, psychiatrists have had a majority of the prescribing and independent practice rights and have been the primary target of pharmaceutical marketing efforts [36]. Nevertheless, PAs and NPs are gaining independent prescribing rights across the country despite the fact that regulations concerning physician-pharmaceutical interactions mainly target physicians [37, 38]. If trends with NPs and PAs follow what this study has found about physicians, policy and regulations concerning pharmaceutical promotions should address all providers, not just physicians.

Our findings provide important context given recent court rulings allowing more flexibility in marketing of off-label drug usage [39]. Our findings indicate the need for both potential policy changes at the time of FDA approval as well as what has been termed “post-market pharmacovigilance” [40]. An effective way to anticipate and control off-label use would be to require information about anticipated off-label use to be presented at the time of a drug’s initial approval review [2]. Furthermore, modification of approval timelines would facilitate more evidence-based review of clinical indications. Current patenting timelines incentivize development of new medications over pursuing new indications for old medications, which further promotes the use of off-label medications rather than putting resources into studying additional indications. The FDA could incentivize a wider range of indications for older drugs via policy changes by granting longer on-patent time for a drug with more studied and approved indications. Post-market pharmacovigilance–the science of collecting, monitoring, researching and evaluating information on adverse drug reactions to identify and prevent harm–should be performed to identify and restrict marketing and promotion of off-label prescriptions that lack robust, peer-reviewed evidence of efficacy [40, 41]. Furthermore, it should be an independent process without industry influence [42]. These policies will protect patient safety and may ultimately drive more evidence-based and cost-effective prescribing choices.

There are limitations to this study that must be considered. While we limited the patient sample to those with only one diagnosis associated with the visit, we acknowledge that this does not necessarily provide the full clinical picture. A patient may have a chronic psychiatric condition that was not the reason for the visit and thus not listed. It is important to note that not all patients in the off-label group were solely receiving off-label medications; some may have been receiving both on- and off-label medications for their diagnosis, and these patients were categorized as among those patients receiving off-label prescriptions. Furthermore, there are times when on-label use indications themselves are also ineffective, and physicians may be willing to try anything that is potentially efficacious [43]. There is an important role for clinicians to provide comprehensive information to patients on anticipated benefits and risks of treatment, especially when prescribing off-label treatment. This is particularly salient for treatment of psychiatric disorders, where anticipated benefits and risks change over time, with differing disease severity, and when alternative treatments may frequently be tried and failed. Furthermore, there are many different psychiatric drugs within the same class, and they are often treated as identical to one another in terms of effectiveness and adverse effects–however, not all have been approved as effective for the same pathologies. A guide to aid clinicians in determining appropriate off-label use has been described and developed [44].

Another limitation is that the study was focused on outpatient visits to psychiatrists for chronic care management and may not be generalizable to psychiatric prescribing among non-psychiatrists, including primary care physicians, or to patients who are hospitalized or presenting with acute psychiatric symptoms. An important point to note is that while these findings are representative of office visits to nonfederal clinics in the United States operating under FDA rules and regulations, they may not be generalizable to non-U.S. settings, where different indications of use may have been approved by regulators or other health authorities. Third, our study was necessarily limited to the use of diagnostic codes to determine which prescriptions were for on-label and off-label indications. There was no way to capture the clinicians’ reasoning for off-label use–perhaps in some cases, the off-label use was well-warranted given undocumented comorbidities or a history of refractory disease that was not improving with traditional, on-label treatment. Alternatively, perhaps the diagnostic code was not issued for that visit, although we did control for this by selecting patients with only one diagnosis associated with the visit. Either way, this study only describes utilization, and cannot examine the risks associated with off-label prescription. Moreover, the NAMCS survey has no information on prescribed dosage, so this information could not be considered when characterizing off-label use Finally, an important predictor that we were not able to examine was the amount and variability of pharmaceutical marketing in our sample. Studies have shown a direct connection between pharmaceutical promotion and physician prescription practice, independent of evidence supporting efficacy of the medication [45, 46], and thus, marketing practices will be an important predictor of off-label use to examine in the future.

## Conclusion

The practice of prescribing drugs for off-label uses can promote innovation in clinical practice, but can also be harmful if not supported by evidence [9]. Examining rates and characteristics of off-label use in specific therapeutic areas, such as psychiatry, and studying predictors of off-label use, can shape future regulatory policy and inform clinical guideline recommendations. We found that off-label prescribing was common, especially for stimulants and anti-psychotics, and that off-label prescription was associated with patient comorbidity and insurance status, as well as clinician type and location. Future studies can and should explore strategies to implement post-market pharmacovigilance in order to minimize adverse drug reactions and ensure patient safety and optimal health outcomes.
